# Prenatal Environmental Stressors and DNA Methylation Levels in Placenta and Peripheral Tissues of Mothers and Neonates Evaluated by Applying Artificial Neural Networks

**DOI:** 10.3390/genes14040836

**Published:** 2023-03-30

**Authors:** Andrea Stoccoro, Vanessa Nicolì, Fabio Coppedè, Enzo Grossi, Giorgio Fedrizzi, Simonetta Menotta, Francesca Lorenzoni, Marta Caretto, Arianna Carmignani, Sabina Pistolesi, Ernesto Burgio, Vassilios Fanos, Lucia Migliore

**Affiliations:** 1 Department of Translational Research and of New Surgical and Medical Technologies, University of Pisa, 56126 Pisa, Italy; 2 Autism Research Unit, Villa Santa Maria Foundation, 22038 Tavernerio, Italy; 3 Istituto Zooprofilattico Sperimentale della Lombardia e dell’Emilia Romagna, Chemical Department, Via P. Fiorini 5, 40127 Bologna, Italy; 4 Division of Neonatology and NICU, Department of Clinical and Experimental Medicine, 56126 Pisa, Italy; 5 Obstetrics and Gynecology Unit 1, Department of Experimental and Clinical Medicine, University of Pisa, 56126 Pisa, Italy; 6 Obstetrics and Gynecology Unit 2, Pisa University Hospital, 56126 Pisa, Italy; 7 First Division of Pathology, Department of Laboratory Medicine, Pisa University Hospital, 56126 Pisa, Italy; 8 European Cancer and Environment Research Institute (ECERI), 1000 Brussels, Belgium; 9 Department of Surgical Sciences, University of Cagliari and Neonatal Intensive Care Unit, AOU Cagliari, 09124 Cagliari, Italy; 10 Department of Laboratory Medicine, Pisa University Hospital, 56126 Pisa, Italy

**Keywords:** epigenetics, DNA methylation, artificial neural networks, DOHaD, placenta, prenatal exposure, heavy metals, dioxins

## Abstract

Exposure to environmental stressors during pregnancy plays an important role in influencing subsequent susceptibility to certain chronic diseases through the modulation of epigenetic mechanisms, including DNA methylation. Our aim was to explore the connections between environmental exposures during gestation with DNA methylation of placental cells, maternal and neonatal buccal cells by applying artificial neural networks (ANNs). A total of 28 mother–infant pairs were enrolled. Data on gestational exposure to adverse environmental factors and on mother health status were collected through the administration of a questionnaire. DNA methylation analyses at both gene-specific and global level were analyzed in placentas, maternal and neonatal buccal cells. In the placenta, the concentrations of various metals and dioxins were also analyzed. Analysis of ANNs revealed that suboptimal birth weight is associated with placental *H19* methylation, maternal stress during pregnancy with methylation levels of *NR3C1* and *BDNF* in placentas and mother’s buccal DNA, respectively, and exposure to air pollutants with maternal *MGMT* methylation. Associations were also observed between placental concentrations of lead, chromium, cadmium and mercury with methylation levels of *OXTR* in placentas, *HSD11B2* in maternal buccal cells and placentas, *MECP2* in neonatal buccal cells, and *MTHFR* in maternal buccal cells. Furthermore, dioxin concentrations were associated with placental *RELN*, neonatal *HSD11B2* and maternal *H19* gene methylation levels. Current results suggest that exposure of pregnant women to environmental stressors during pregnancy could induce aberrant methylation levels in genes linked to several pathways important for embryogenesis in both the placenta, potentially affecting foetal development, and in the peripheral tissues of mothers and infants, potentially providing peripheral biomarkers of environmental exposure.

## 1. Introduction

The Developmental Origins of Health and Disease (DOHaD) theory provides a better explanation than any other model of the continued rise in chronic noncommunicable disease pandemics [1]. It derives from investigations conducted in the late 1980s by a British paediatrician and epidemiologist, David Barker, who realized that the continuous increase in cardiovascular and metabolic pathologies was linked to specific conditions of foetal life. The developmental periods are currently considered as “windows of sensibility”, as capable of shaping how the growing organism will respond to future challenges. In particular, intrauterine growth retardation (IUGR) condition frequently determines long-term effects on the metabolism not only of the foetus and the child, but also of the adult, giving rise to obesity and diabetes type 2 [2]. IUGR, as well as newborn overgrowth, are conditions that arise from different kinds of stimuli, mainly mediated by the in utero environment. Alcohol, tobacco and drugs during pregnancy, or early exposure to phthalates, dioxins, heavy metals or endocrine-disrupting chemicals are often associated with an increased risk of gestational and foetus complications, including metabolic or neurodevelopmental impairment [3,4,5].

It is now established that exposure to environmental stressors during pregnancy plays an important role in influencing susceptibility to certain chronic diseases later in life through the modulation of epigenetic mechanisms, including DNA methylation [6,7,8].

Therefore, it is of utmost importance to better understand the relationship between prenatal exposure to adverse environmental factors and changes in epigenome. In this way, the placenta is crucial in mediating the effect of the environment on the child health development programming, as it orchestrates the development and regulation of the foetal environment [9]. Indeed, several studies conducted in humans and rodents have linked various prenatal factors, including maternal psychosocial stress, maternal smoking and exposure to air pollutants, metals and chemicals, to the placental DNA methylation and gene expression levels, as well as to infant and childhood health outcomes [10,11,12,13]. From these studies, a plethora of critical genes involved in different cellular pathways, such as growth, metabolism or neuronal plasticity, have been identified as epigenetically modulated. The epigenetic deregulation of some of them, such as the *IGF2, H19* and *LEP* genes, has been frequently investigated in foetal-derived tissues following different types of exposure and often correlated to poor pregnancy conditions [14,15,16,17,18,19,20]. Other candidate genes, including *CYP1A1*, *COMT*, *NR3C1*, *BDNF*, *ER-α*, *OXTR* and *HSD11β2*, exhibited altered methylation and expression in placenta, cord blood or in infant buccal cells, linked to environmental pollutants, maternal smoking or depression [21,22,23,24,25,26,27].

To date, most of these studies have evaluated the contribution of one or a few environmental exposures to global or gene-specific methylation levels, but how various environmental factors interact with each other is still poorly understood [12]. Indeed, different environmental factors in nature likely act in concert in inducing epigenetic alterations, and the investigation of these interactions is of fundamental importance to better understand the complexity of the process. In this context, artificial neural networks (ANNs) are a solid method to overcome the intrinsic limitations of standard statistical methodologies to investigate non-linear associations that characterize complex biological systems, and are successfully applied in genomics and epigenomics research [28,29,30]. In particular, the auto-contractive map (Auto-CM) is a powerful data mining system able to define the strength of the association of each variable with all the others and to visually show the map of their main connections. The added value of this approach in investigating biological systems is represented by its ability in evidencing the organizing principles of a network of variables and therefore to map biological processes using automatic and analytical models to reconstruct the imprecise, nonlinear and simultaneous pathways, underlying a complex set of data. In recent years, Auto-CM has been extensively applied to unravel the complexity of connections among environmental, clinical and biological variables in several medical datasets, leading to the identification of genetic and epigenetic biomarkers for neurodevelopmental disorders, cancer and lifestyle outcomes [31,32,33].

In our mind, there is the development of a study model that could relate maternal–foetal exposure to environmental factors (such as heavy metals and potentially epigenotoxic organic molecules) with the continuous increase in endocrine–metabolic pathologies, neurodevelopmental disorders, immune-mediated and tumor pathologies through a multidisciplinary approach. The fundamental design idea was to pave the way for research on different exposure conditions for the mother and foetus dyad, collect specific biomarkers related to early exposure and prospectively provide a follow-up in the coming years. Specifically, in the present study we aimed to explore the connections between various prenatal exposures and DNA methylation in the placenta and in maternal and neonatal buccal cells. To this end, we enrolled mother–infant pairs, assessed prenatal maternal exposures through a questionnaire, measured the concentrations of dioxins and heavy metals in the placenta, and analyzed both global methylation and hydroxymethylation (the global levels of 5-methylcytosine and 5-hydroxymethylcytosine: 5-mC and 5-hmC content, respectively) and the gene-specific methylation levels of several imprinted, metabolic and/or neurodevelopmental genes (*LEP*, *MECP2*, *IGF2*, *MTHFR*, *DNMT3B*, *OXTR*, *H19-ICR*, *HSD11B2*, *BDNF*, *CYP1A1*, *ERα*, *MGMT*, *RELN*, *NR3C1* and *COMT*) in the DNA obtained from the placenta and buccal mucosa cells of mothers and newborns. We then applied Auto-CM to unravel the connections among the investigated environmental factors and DNA methylation marks.

## 2. Materials and Methods

### 2.1. Study Population

Between February 2019 and February 2020, 28 pregnant women were recruited at the Units of Obstetrics and Gynaecology, Santa Chiara Hospital in Pisa. The clinical characteristics and anthropometric measurements of each newborn/mother couple are shown in Table 1. 

To standardize the delivery features, exclusively elective caesarean deliveries without labour or general anaesthesia were included. Moreover, in order to exclude confounding factors known to affect DNA methylation patterns, a wide range of exclusion criteria were established. They included obesity, pre-existing diabetes, autoimmune diseases (e.g., thyroiditis), current multiple pregnancies, foetal chromosomopathies, alcohol or drug abuse, and other characteristics summarized in Table 2.

The eligibility of the subjects was established after the administration of a questionnaire, during the medical exam scheduled to plan the delivery date. The aim of the questionnaire was to explore the periconceptional and gestational periods, in order to establish the influences occurred, in term of professional or environmental exposures (e.g., chemicals, solvents, dioxins or air pollutants) and maternal characteristics and stressors. Maternal stress factors that could insult the neurodevelopment were previously investigated by our group, by using the same questionnaire [34]. Furthermore, data on parental lifestyle and anthropometric measurements of newborns at birth were collected. In particular, we collected the following information: sex of the infants, birth weight, body length, head circumference, weight of the placenta, gestational age and eventually neonatal complications.

Informed written consent was signed by parents who consented to contribute to the project, and the sampling and experimental processes were performed with the approval of Area Vasta Nord Ovest Ethics Committee (CEAVNO) (study protocol number 40896; approved on 19 July 2018).

### 2.2. Samples Collection and DNA Extraction

For each mother–newborn couple, samples of buccal cells were collected within 48 h from childbirth. Cells were collected easily and quickly using a DNA Buccal Swabs Kit (Isohelix, Kent, UK) by gently swabbing the swab on the inner walls of the cheeks, following the instructions of the company. The addition of Isohelix Dri-Capsules preserved, at room temperature, genomic DNA from degradation after collection.

The placenta samples were collected within one hour of childbirth following standard obstetrical procedures. The placenta was delivered and immediately transferred in a sterile container and weighed. First, a macroscopic anatomopathological examination to verify the health of the organ was carried out. In order to prevent contamination from the maternal skin and/or surrounding environment, the maternal decidua was discarded and only the inner part of the placenta was retained. Subsequently, four placenta sections of about 1–2 cm^3^ were excised from the maternal side of the placenta and washed several times in Phosphate Buffered Saline (PBS (Gibco—ThermoFisher, Waltham, MA, USA)) to remove the presence of maternal blood. Placenta and umbilical cord samples for each dyad were also collected, in order to send samples to other collaborating units, to allow measurement of the toxic compounds. Finally, the tissue samples were stored at −80 °C for further analysis. Placental genomic DNA was extracted using the QIAamp^®^DNA Mini and Blood Mini Handbook Kit (QIAGEN, Milan, Italy), following the manufacturers’ protocol and stored at −20 °C until use. 

### 2.3. Study Questionnaire

A questionnaire previously detailed by us was administered to the women enrolled in the study [34]. We collected information on maternal age, pre-pregnancy body mass index (BMI), gestational age and weight of the babies at birth. Moreover, data on maternal environmental exposure (e.g., occupational exposure to chemicals or solvents), health status, lifestyle during pregnancy (e.g., tobacco smoking), stressful events (e.g., death of relatives, loss of work, divorce, legal problems) and episodes of fever or flu were acquired. Table 3 shows the distribution of data collected by the administration of the questionnaire.

### 2.4. DNA Methylation Analyses (HRM and Global Methylation)

Preliminarily, 200 ng of DNA derived from each tissue was bisulfite treated using the “EpiTect^®^ Bisulfite Kit” (QIAGEN, Milan, Italy). Reaction with sodium bisulfite promotes cytosine deamination and its conversion into uracil; 5-methylcytosine (5-mC)—the methylated form of the cytosine—is resistant to deamination by bisulfite treatment, so that bisulfite treatment converts unmethylated cytosines leaving methylated cytosines unchanged.

Gene-specific methylation was performed using the methylation sensitive–high resolution melting (MS-HRM) technique with a CFX96 Real-Time PCR detection system (Bio-Rad, Milan, Italy). In-house MS-HRM protocols, using methylation-independent primers designed with the MethPrimer software were designed. Each couple of primers was first tested by a Gradient PCR in order to identify the optimal annealing temperature (Ta). Details on primer sequences and Ta used during MS-HRM analysis are shown it Table 4. For MS-HRM analysis, a bisulfite DNA amplification was first performed through a PCR phase that included an initial step at 95 °C for 3 min, followed by 60 cycles of 95 °C for 30 s, a specific Ta for each gene as reported in Table 4 for 30 s and 15 s at 72 °C. The PCR was followed by an HRM step that included a denaturation step (10 s at 95 °C), a renaturation step (1 min at 50 °C) and then a rise in temperature from 65 °C to 95 °C, with an increment of temperature of 0.2 °C every 15 s. MS-HRM analyses were carried out with reactions that included 5 μL of master mix (BioRad, Milan, Italy), 10 pmol of the forward and reverse primers and 10 ng of DNA samples previously treated with sodium bisulfite. 

Standard DNA samples with known methylation percentages were prepared by mixing fully methylated and unmethylated DNA (EpiTectH methylated and unmethylated human control DNA, bisulfite converted, Qiagen). Six growing ratios of methylation were thus obtained (0, 12.5, 25, 50, 75 and 100%) and were used to generate standard values to be inserted in an interpolation curve that allowed to obtain specific methylation levels of each sample, as previously detailed [35].

Global DNA methylation was assessed by means of the MethylFlashTM Methylated DNA Quantification Kit (Colorimetric) (Epigentek Group Inc., New York, NY, USA) and the MethylFlashTM Hydroxymethylated DNA Quantification Kit (Colorimetric) were used to detect the global methylation status of DNA isolated from all tissue samples.

These methods allow the detection and quantification of methylated 5-mC and 5-hmC DNA, respectively, using optimized antibodies and enhancing solutions with high specificity toward them. The experiments were conducted according to production protocols. 

### 2.5. Evaluation of Metals and Dioxins Concentrations in the Placenta

The metals’ evaluation was carried out using an Inductively Coupled Plasma Mass Spectrometer (ICP-MS). High-purity deionized water was purchased by Evoqua water technologies (Barbsbuttel DE), the nitric acid was sourced from J.T. Baker (Center Valley, PA, USA) and hydrochloric acid was obtained from Sigma (St. Louis, MO, USA). About 3 g of each sample was homogenized and added to 10 mL of nitric acid in a 50 mL polypropylene screwcap tube (Digi-Tubes SCP Science). Specimens were placed in a Digi-Prep system (SCP Science) at 75 °C overnight. Following cooling, samples were diluted with water to a final volume of 20 mL. One more dilution (10 times) with a solution including nitric acid 2% and hydrochloric acid 1% (*v/v*) was carried out before further analyses. The ICP/MS (7700 Agilent Technologies, Santa Clara, CA, USA) with an ASX-500 CETAC Autosampler (Cetac Technologies, Omaha, NE, USA) was employed to perform sample analyses. A solution of bismuth (1 μg/mL) was used as the internal standard to perform the analyses. Briefly, the instrument conditions were as follows: RF power 1550 W; RFmatching 1.8 V; carrier gas (He) 1.7 L min. The analyses were carried out with MassHunter 4.2 Software (Agilent Technologies) and the quantitative determination was performed on the most abundant and least interfered isotope.

Dioxins (PCDD/Fs) and polychlorinated biphenyls (PCBs) were determined with the HRGC/HRMS methods that were a modification of the US EPA Method 1613/B (1994) and US EPA Method 1668/C (2010). The modifications were needed to adapt these methodologies to the matrix analyzed and to determine the concentration of all congeners at background and interest levels. The analysis was performed by means of 13C–labelled internal standards and measurement was carried out with Gas Chromatography—High Resolution Mass Spectrometry (HRGC-HRMS). Samples were homogenized and freeze-dried (Freeze Dryer Martin Christ, Germany) before the analysis. In this phase, specimens underwent numerous cycles of medium and high vacuum and low temperature to remove the large amount of water. Samples were further homogenized after freeze-drying, and a portion was used for the determination of the lipid content using the Soxhlet method. The LoQ values for PCDD/Fs + DLPCBs were in the range 0.005–0.04 pg TEQ/g. The analytical results were presented as “upper bound” levels (detected lower or equal to the LoQ) using the toxic equivalency factors proposed by the World Health Organization in 2005 [36].

### 2.6. Artificial Neural Networks

The connections among the variables were obtained by means of an artificial adaptive system named Auto-CM, which is a special kind of artificial neural network (ANN) able to develop weights that are a function of the strength of the associations. The weights are then translated in physical distances so that the variables whose connection weights are higher become nearer and vice versa. Following the training phase, the weights generated by the Auto-CM depict the warped landscape of the variables. Then, for the weights matrix of the Auto-CM system, a simple filter was employed in order to obtain a map of the main connections between the variables of the dataset and the basic semantics of their similarities. This process defined the connectivity map [37].

Data were stratified according to the categories shown in Table 3. For each class, a binary code was applied (0 if variable absent; 1 if variable present). Gene-specific, global DNA methylation levels, metals and dioxins/polychlorobiphenyls dioxin-like (PCBDL) concentrations were transformed into input variables scaled from 0 to 1. As an example, the placenta *H19* methylation variable had methylation values that ranged from 40.7% (lowest observed value) to 76.1% (highest value). The transformation, methylation level of 40.7 (the lowest value) became 0, and the methylation levels of 76.1 (the highest value) became 1, and consequently the other placenta *H19* methylation values were scaled in accordance with this new range. The projection of the placenta *H19* methylation in the map shows the position of placenta *H19* methylation according to its high values. The same transformation was used for the methylation levels of the other genes investigated and for global methylation and hydroxymethylation levels. This data transformation is usually performed to analyze data in ANN investigations [37]. The distribution of DNA methylation and hydroxymethylation levels in the study cohort is shown in Table 5. The distribution of metals and dioxin/PCBDL placenta concentrations is shown in Table 6.

## 3. Results

**Figure 1:**
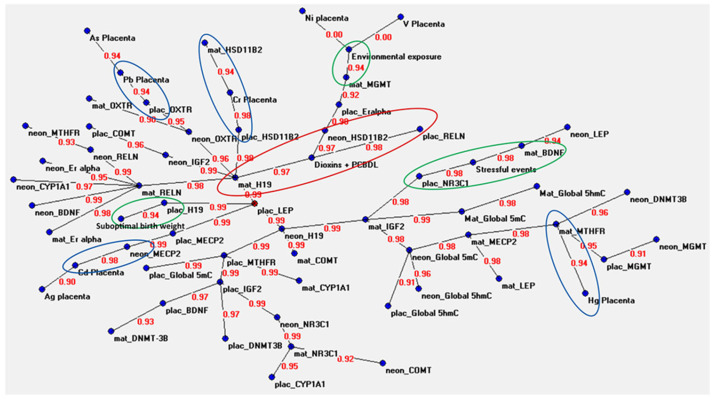
Semantic connectivity map obtained with the auto-contractive map system. The values between two variables correspond to the strength of the associations between them. These values range from 0 (which means that the variables are not connected) to 1 (which means that the variables are highly connected). The green circles indicate relevant connections between DNA methylation and questionnaire data. The blue circles indicate relevant connections between DNA methylation data and metals in the placenta. The red circle indicates relevant connections between DNA methylation data and dioxins/PCBs in the placenta.

The connections among in utero exposure to various environmental stressors and gene-specific and global methylation levels, in DNA from the placenta and from mother and infant buccal swab cells, were investigated by applying Auto-CM. Figure 1 shows the graph of the ANN analysis, i.e., the semantic connectivity map showing the connections between the analyzed variables and the strength of each connection. Each variable is indicated with a blue dot (node), except for placental *LEP* methylation that is indicated with a red dot as it represents a hub of the system (a variable that is connected with several other variables). Lines indicate connections between variables, and the red number on each line, ranging from 0 to 1, indicates the strength of the association (SA). When the SA is equal or close to 1, it means that the two variables are highly connected; by contrast, a SA of 0 or close to 0 means no association or a low association.

Most of the observed connections were among gene-specific methylation, and global 5-mC and 5-hmC levels in the investigated tissues. For example, the placental methylation levels of the *LEP* gene (leptin) showed a strong association with the methylation levels of the *H19* gene evaluated in the maternal mucosa (SA = 0.99), with the methylation levels of the *H19* gene evaluated in the neonatal mucosa (SA = 0.99), with the methylation levels of the *H19* gene in the placenta (SA = 0.99) and with the methylation levels of the *MECP2* gene in the placenta (SA = 0.99). All these variables were in turn connected with the methylation levels of several other genes, some of which connected with certain environmental factors (Figure 1). Some interesting connections were observed among placental, neonatal mucosa and maternal mucosa *OXTR* gene methylation levels (SAs ranging from 0.90 to 0.95), between the maternal and neonatal mucosa methylation levels of the *RELN* gene (SA = 0.99), between global 5-mC and 5-hmC levels in both maternal (SA = 0.99) and neonatal (SA = 0.96) buccal mucosa cells, as well as between maternal *H19* and neonatal *IGF2* methylation levels (SA = 0.99) and between maternal *IGF2* and neonatal *H19* methylation levels (SA = 0.99) in buccal mucosa cells.

Concerning the connections among DNA methylation data, environmental or anthropometric factors and the levels of metals and dioxins in the placenta, we highlighted some of the most interesting associations using different coloured circles in Figure 1.

With regard to the connections among DNA methylation, prenatal exposures assessed through questionnaire and the measured anthropometric data of newborns (highlighted by green cycles in Figure 1), the most interesting connections were observed between environmental exposures during pregnancy and the methylation levels of the *MGMT* gene in the maternal buccal mucosa (SA = 0.94), between stressful events and both *BDNF* methylation in the maternal buccal mucosa (SA = 0.94) and *NR3C1* methylation in the placenta (SA = 0.98), and between *H19* gene methylation in the placenta and suboptimal birth weight (binding strength = 0.94).

Associations among DNA methylation levels and the measured levels of metals and dioxins/PCBs in the placenta are highlighted in Figure 1 by blue and red cycles, respectively. Auto-CM revealed strong associations between the placental concentrations of mercury and the methylation levels of the *MTHFR* gene in the maternal buccal mucosa (SA = 0.94), of cadmium and methylation of the *MECP2* gene in the newborn buccal cells (SA = 0.99), of lead and methylation of the *OXTR* gene in the placenta (SA = 0.94), of chromium and methylation of the *HSD11B2* gene in both maternal buccal mucosa (SA = 0.94) and placenta (SA = 0.98), and of dioxins/PCBs and the methylation levels of *RELN* in the placenta (SA = 0.98), of *HSD11B2* in the newborns buccal cells (SA = 0.97), and of *H19* in the maternal buccal mucosa (SA = 0.97).

## 4. Discussion

In the current study, we explored the connections among environmental exposures during gestation with DNA methylation levels detected in placentas, as well as in maternal and neonatal buccal cells from 28 mother–infant couples by applying ANNs. ANN analysis revealed that the majority of the connections were among global DNA methylation, global hydroxymethylation and DNA methylation patterns of the genes evaluated in placentas, maternal buccal cells and neonatal buccal cells. ANNs also revealed that suboptimal birth weight was associated with placental *H19* methylation, maternal stress during pregnancy with placental *NR3C1* and maternal *BDNF* methylation as well as exposure to air pollutants with maternal *MGMT* methylation. Associations among placental concentrations of lead, chromium, cadmium and mercury with placental *OXTR*, maternal and placental *HSD11B2*, neonatal *MECP2* and maternal *MTHFR* methylation levels, respectively, as well as among concentrations of dioxins and placental *RELN*, neonatal *HSD11B2* and maternal *H19* methylation levels were also observed.

ANN identified the placental methylation levels of the *LEP* gene as the mathematical centre of the semantic connectivity map. The *LEP* gene encodes a protein hormone that is secreted by adipocytes into the circulation and plays an important role in the regulation of energy homeostasis [38]. In the placenta, leptin is a homeostatic regulator that promotes the proliferation, protein synthesis and expression of tolerogenic maternal response molecules and exerts an anti-apoptotic action by controlling the expression of p53 under different stress conditions [38]. Placenta leptin expression is regulated by several molecules, including human chorionic gonadotropin, steroids, insulin and other growth-linked hormones, suggesting that it may have a pivotal endocrine function in the trophoblast [38]. Interestingly, ANNs showed that placental *LEP* gene methylation was strongly associated with *H19* methylation evaluated in all the three different tissues analyzed, i.e., placenta, maternal and neonatal buccal cells. The *H19* gene, which is among the most expressed genes in placenta, encodes a long-non-coding RNA that plays a key role in placental development [39]. The *H19* gene is located within an imprinted genomic locus and is predominantly expressed by the maternal chromosome. Alterations in the imprinting of *H19* and the *IGF2* genes can lead to abnormalities of placental and foetal growth and are associated with Silver–Russell and Beckwith–Wiedemann syndromes, which are characterized by severe growth restriction and overgrowth, respectively [39]. It is therefore interesting to note that from the analysis of ANNs, a close association emerges between the methylation of the *LEP* gene, a gene important for placental growth, and the methylation of the *H19* gene, whose altered imprinting is associated with developmental diseases, in all the three types of samples examined. To the best of our knowledge, such association has never been reported in the literature. Furthermore, ANNs showed a close association between suboptimal birth weight and methylation of the *H19* gene in the placenta (discussed in depth below), further highlighting how the methylation of the *H19* gene could play a fundamental role in foetal development. Placental *LEP* gene methylation was also strongly associated with *MECP2* gene methylation. The *MECP2* gene (methyl CpG binding protein 2) encodes a protein involved in the “reading” of epigenetic marks, and in particular, in the recognition of the methylated cytosines to which it binds, inducing the transcriptional repression of the associated gene. The *MECP2* gene plays a fundamental role in neurodevelopment and mutations in this gene are causative of Rett syndrome. The *MECP2* protein is also involved in the control of body weight by modulating the pathway that controls the expression of leptin through post-translational modifications [40,41]. Results of the present ANNs analysis show for the first time a relationship between the methylation levels of the two genes, suggesting that this epigenetic modification could also be involved in the regulation of leptin by *MECP2*.

The association between suboptimal birth weight and placental *H19* methylation is of particular interest given the pivotal role that *H19* expression in placenta plays in foetal development. Already, Burque and coworkers noted in 2010 that the 11p15.5 ICR1 (associated with *H19* and *IGF2*) methylation was characterized by a marked intra-placental variability, finding that a decrement in the *H19/IGF2* imprinting control region placental methylation was associated with intrauterine growth restriction but not with preeclampsia [42]. A following study detected that increased methylation of the *H19/IGF2* ICR on placenta samples positively correlated with the establishment of the newborn’s weight, independently from maternal diabetic status, glucose concentrations or prenatal maternal body mass index [43]. On the other hand, a following study observed that the macrosomia induced by intrauterine hyperglycemia was strongly associated with the cord blood methylation and placenta gene expression status of *IGF2/H19* [44]. A deregulation of *H19* expression was also observed in the placenta of infants small for gestational age (SGA) [45], confirming the important role of this gene for foetal growth. However, the results in the literature regarding *H19* gene methylation and foetal growth are not unique. For example, an increased methylation of the *H19* gene was observed in the DNA extracted from the peripheral blood of both small and large infants for gestational age compared to normal infants for gestational age [46]. In another study, no differences in *H19* gene methylation levels measured in peripheral blood were observed between normal and SGA infants [47], and similarly, no altered methylation was observed in placental tissue in the first trimester of gestation obtained from chorionic villus biopsies [48]. On the other hand, a positive correlation between the hydroxymethylation levels of the *H19* gene in the placenta and birth weight has been observed by other authors [49] and *H19* expression was found to be significantly decreased in SGA placentas after 34 weeks of gestation without pregnancy complications and abnormal histopathological morphology [50]. Although the role of *H19* in the foetal development remains unclear, results of the present study seem to confirm its involvement in foetal growth and suggest that its function can be modulated by its methylation levels.

Of particular interest is the observation that maternal stress during pregnancy is strongly associated with placental *NR3C1* and maternal *BDNF* methylation levels. The *BDNF* gene encodes the brain neurotrophic factor, which is essential for the growth, differentiation and survival of neurons. The *NR3C1* gene encodes the glucocorticoid receptor which plays a vital role in regulating genes that control development, metabolism and immune response. Altered methylation of the *BDNF* gene was found in the placental tissue, in the umbilical cord and venous blood of women who suffered traumatic events during pregnancy [51], while altered methylation of the *NR3C1* gene was found in the DNA extracted from the umbilical cord of newborns born to depressed mothers [52]. Moreover, altered methylation of both genes has already been associated with stressful events in pregnancy in the buccal mucosa of newborns at 2 months [23]. Therefore, results highlighted by ANNs confirm previous results in the literature, suggesting how the methylation levels of the *BDNF* and *NR3C1* genes are highly sensitive to maternal stress.

ANNs also revealed that environmental exposure is associated with maternal *MGMT* methylation. The information regarding environmental exposures was provided by the mothers in completing the questionnaire and refers to any environmental/occupational exposure to pollutants during the pre-pregnancy and pregnancy period. It is well known that exposures to environmental pollutants have an effect on DNA methylation, and alterations at the level of the *MGMT* gene have already been observed in the DNA extracted from the peripheral blood of workers exposed to benzene [53,54], of individuals exposed to pesticides [55] and of individuals exposed to diesel engine exhaust [56]. However, this is the first time that an association has been observed between exposure to environmental pollutants and altered methylation of the *MGMT* gene in the maternal buccal mucosa. This finding could be of considerable interest considering that the *MGMT* gene encodes a DNA repair enzyme involved in cellular defense against mutagenesis and toxicity from alkylating agents and that its altered methylation in saliva has been associated with the risk of developing tumours of the head and neck [57].

ANNs revealed that placenta concentrations of lead, chromium, cadmium and mercury were associated with placental *OXTR*, maternal and placental *HSD11B2*, neonatal *MECP2* and maternal *MTHFR* methylation, respectively. Some works in the literature have shown how prenatal exposure to metals induces alterations in DNA methylation in various tissues. For example, the concentration of several metals including arsenic, cadmium, lead, manganese and mercury in children’s nails has been associated with increased placenta methylation of the *NR3C1* gene [58], while the cadmium concentration in the placenta has been associated with alterations in the methylation of DNA extracted from the placenta in numerous genomic loci [59]. Furthermore, in a 2015 study, 339 gene loci were identified whose placental methylation was associated with prenatal mercury exposure, assessed by measuring the concentration of mercury in children’s nails [60]. In this latter study, authors also associated the methylation changes of these loci with a high-risk neurodevelopmental profile. The results obtained from ANNs confirm that the presence of metals is associated with the altered methylation of various genes at placental level, maternal and neonatal buccal cell DNA. In particular, it is interesting to note how the methylation of the *HSD11B2* gene is strongly associated with the presence of chromium in the placenta, both at the level of placental tissue and at the level of the maternal buccal mucosa, suggesting that this latter evaluation can be used as a peripheral biomarker of exposure to metals. It is well known that the presence of heavy metals in the placenta could induce adverse effects in newborns. For example, placental levels of cadmium, lead and arsenic have been found to negatively correlate with low birth weight [61].

ANNs also revealed interesting associations between dioxins/PCB placenta concentrations and placental *RELN*, neonatal *HSD11B2* and maternal *H19* methylation. It is interesting to note that these pollutants, in addition to having effects on placental DNA methylation (*RELN* gene), also have effects on DNA extracted from the mucosa of newborns (*HSD11B2* gene) and of mothers (*H19* gene), suggesting also in this case their possible use as a peripheral biomarker of prenatal exposure to these pollutants.

We are not far from the possibility of identifying specific biomarkers (e.g., epigenetic alterations of certain genes) related to specific environmental exposures; for instance, after developmental exposure to Pb, altered CpGs methylation at imprinted loci was found in mice [62], and in the Michigan Mother and Infant Pairs Cohort, 38 CpG sites were found to be significantly associated with maternal bisphenol A exposure (urinary bisphenols) and the gene-set having the greatest odds of enrichment for differential methylation was type I interferon receptor binding [63]. More recently, in a mouse model of exposure to a human-relevant mixture of polychlorinated biphenyls, it was observed that placenta and foetal brain displayed shared DNA methylation alterations at regions related to neurodevelopment and autism spectrum disorders [64].

To the best of our knowledge, this is the first investigation that evaluated the associations between DNA methylation, at both global and gene-specific levels, with adverse environmental factors collected with the administration of a questionnaire to mothers, and with the concentrations of various metals and dioxins/PCB in the placenta in a well-defined mother–infant cohort. Moreover, this is the first study that concurrently evaluated DNA methylation in both placenta specimens and in the buccal mucosa cells of the mothers and infants. The present results suggest that the simultaneous evaluation of DNA methylation in placenta and in mother and infant buccal cells in association with several adverse environmental factors could provide new insights into the developmental pathways affected by that exposure, as well as peripheral biomarkers. Further investigations with a similar study design, with a higher number of mother–newborn couples and/or with DNA methylation analyses at the genome-wide level are needed to confirm current results and/or to find new DNA methylation biomarkers of exposure to adverse environmental factors during pregnancy. Indeed, one of the limits of the current study is the analysis of a limited number of genes. We selected them in the literature as they are involved in neurodevelopment (*MECP2*, *OXTR*, *RELN*), DNA methylation (*MTHFR*, *DNMT3B*), regulation of metabolism (*LEP*), fetus growth (*IGF2*, *H19*), placental cortisol regulation (*HSD11B2*, *NR3C1*), DNA repair (*MGMT*), xenobiotic metabolism (*CYP1A1*), hormonal metabolism (Erα) and neural activity (*BDNF*, *COMT*), all pivotal pathways for fetus development. However, the methylation of several other genomic loci could be associated with the variables considered in a similar manner observed here or with stronger effects. Moreover, the selection of two imprinted genes in the current study, including *IGF2* and *H19*, could introduce some bias in the associations, as the methylation of imprinting control regions is established in the early stages of embryogenesis, is maintained mitotically and plays a pivotal role in placenta functioning [65]. There is evidence that imprinted gene methylation levels are also sensitive to adverse environmental factors, and methylation changes in imprinting control regions have been associated with diseases of foetal origin in adult individuals [45,66]. However, the sensitivity of DNA methylation modifications due to environmental exposure could be different in imprinted and non-imprinted genes, given that the methylation of the former is already established during germline development and maintained during embryogenesis, and could have different effects on foetal development [67]. Future studies that analyze the association between adverse environmental factors and DNA methylation levels both in imprinted and non-imprinted genes could clarify whether these genes could act as differential targets.

## 5. Conclusions

In conclusion, results from the present study suggest several potential connections between in utero exposures and DNA methylation levels in placental maternal and neonatal tissues. We are aware of the limits of the present study in terms of sample size and monocentric nature of the study. Despite the Auto-CM system that we employed proved to be able to find reliable connections even in small datasets [31,32,33,34,37,68,69,70], replication of the present findings in larger cohorts is required both to confirm the observed associations and to test for the contribution of geographic, ethnic, genetic and region-specific environmental factors.

## Figures and Tables

**Table 1:** Characteristics of the study cohort.

Mothers		Newborns	
Number	*n* = 28	Males	*n* = 17
Age (years)	34.10 ± 4.43	Females	*n* = 11
Pregravid BMI (Kg/m^2^)	21.77 ± 2.63	Gestational age (days)	38.88 ± 0.48
Previous pregnancies	92%	Weight (g)	3247.42 ± 382.45
Previous miscarriages	19%	Length (cm)	50.07 ± 1.84
Placental weight (g)	587.96 ± 134.11	Head circumference (cm)	35.14 ± 1.43
		Apgar score	>7

**Table 2:** Inclusion and exclusion criteria.

Inclusion Criteria	Exclusion Criteria
Italian nationality	Family history of metabolic disorders (e.g., gestational diabetes, autoimmune diseases)
Age (from 18 to 40 years)	Preeclampsia
Pre-pregnancy BMI 18.5–25 kg/m^2^	Twin pregnancy
Ability to understand the study protocol and willingness to accept it	Chromosomopathies
Propensity to breastfeed	Maternal infections
Singleton birth	Pharmacological treatments
Natural pregnancy	Drugs or alcohol abuse
Elective caesarean delivery	Labour
Gestational age ≥ 34 weeks	General anesthesia

**Table 3:** Distribution of maternal and newborn data from the study questionnaire.

Parameter	Outcome	
Birth weight	Suboptimal (2500–3000 g)	Adequate (3000–4000 g)
	(*n* = 6) 21.4%	(*n* = 22) 78.6%
Stressful events during pregnancy	Yes	No
	(*n* = 9) 32.2%	(*n* = 19) 67.8%
Maternal age at birth	<40 years old	≥40 years old
	(*n* = 25) 89.3%	(*n* = 3) 10.7%
Environmental exposure	Yes	No
	(*n* = 3) 10.7%	(*n* = 25) 89.3%
Fever or flu during pregnancy	Yes	No
	(*n* = 8) 28.6%	(*n* = 20) 71.4%
Living context	Rural	Urban
	(*n* = 9) 32.2%	(*n* = 19) 67.8%
Smoking before conception	Yes	No
	(*n* = 7) 25%	(*n* = 21) 75%
Exposure to passive smoking	Yes	No
	(*n* = 6) 21.4%	(*n* = 22) 78.6%

**Table 4:** Main characteristics of the designed primers. Annealing temperature (Ta), amplicon size and number of CpG sites included in the analyzed amplicons are reported.

Gene	Primer Sequence	Ta	Amplicon Size	CpG Sites	Accession Number and Nucleotide Position
*LEP*	F 5′-GGGTGGGATTTTAGAATTTTTAATT-3′ R 5′- AAACCAACCCCTTAAAAAAATACTT-3′	55°	259 bp	28	NG_007450. From 14444 to 14703 bp
*MECP2*	F 5′-AATTAAGGTTTTTTAGTTGGGGTAA-3′ R 5′-TTAACCCTCTATCCACAAATACACC-3′	62°	145 bp	5	NC_000023.11 From 154097160 to 154097350 bp
*IGF2*	F 5′- GGAGGGGGTTTATTTTTTTAGGAAG -3′ R 5′- AACCCCAACAAAAACCACTAAACAC-3′	60°	93 bp	3	NG_008849.1 From 6281 to 6374 bp
*MTHFR*	F 5′-TTTTAATTTTTGTTTGGAGGGTAGT-3′ R 5′-AAAAAAACCACTTATCACCAAATTC-3′	54°	155 bp	7	NM_005957.4 From 30 to 184 bp
*DNMT3B*	F 5′-TGGTGTTGTGTGATTATAGTGG-3′ R 5′-TCACCCTAAAAAATCAAAAACC-3′	55°	174 bp	6	NG_007290.1 from −397 to −223 bp
*OXTR*	F5′-AATTATTGTAAAATAAATTTATTTGTTAAG-3′ R 5′-AACTAAAATCTCTCACTAAAACCTC-3′	53°	274 bp	26	NC_000003.11 From 8812437 to 8812711 bp
*H19*	F 5′-TGGGTATTTTTGGAGGTTTTTTT-3′ R 5′-ATAAATATCCTATTCCCAAATAA-3′	56°	216 bp	17	NG_041945.1 From 3549 to 3764 bp
*HSD11B2*	F 5′-TAGGTTTAAGTTTTGGAAGGAAAG-3′ R 5′-ACCACAAAACCTACCTAAAACAAAA-3′	59°	107 bp	5	NG_016549.1 From 4302 to 4409 bp
*BDNF*	F 5′-GGGTTGTTAATTTATATTTGGGAAGT-3′ R 5′-ACCACTAATTACCCACAAAACC-3′	58°	119 bp	4	CM000673.2 From 46058 to 46177 bp
*CYP1A1*	F 5′-TGTTATAGGGTTTTTAGGAAAAA-3′ R 5′-AAATTATTTTCTAACCTAAACCAAC-3′	54.8°	147 bp	4	GRCh37/hg19 From 75013061 to 75017877 bp
*Erα*	F 5′-GGGAGATTAGTATTTAAAGTTGGAGGT-3′ R 5′-CAAAACAAAAAACTCAAAAACC-3′	55.4°	233 bp	22	NG_008493.2 From 155951 to 156184 bp
*MGMT*	F 5′-GCGTTTCGGATATGTTGGGATAAGT -3′ R 5′-AACGACCCAAACACTCACCAAA -3′	58°	110	12	NC_000010.11 From 129467205 to 129467315 bp
*RELN*	F: 5′-TTGAAGAGTTTAGAAGTAATGAATAATAGA-3′ R: 5′-ACCTCATCTATAAAAAATTTTAAAATAAAA-3′	56 °C	192	7	NG 011877.2 (4053–4244)
*NR3C1*	F 5′-TTTTATAAAAATTTTTTTGGTTGAGG -3′ R 5′-TAAACTTTCAACAAACCTCTTATCC -3′	54°	167	9	NG_009062.1 From 33776 to 33943
*COMT*	F 5′-GTTTATGGGTGATATTAAGGAGTAG -3′ R 5′-AATAAATATCAATAACCTCCAACAC -3′	55°	101	6	NG_011526.1 From 25933 to 2634

**Table 5:** Distribution of both gene-specific methylation and global methylation and hydroxymethylation levels in the placenta, neonatal mucosa and maternal mucosa expressed as mean ± standard deviation.

Investigated Genes	DNA Methylation Levels (%, Mean ± Standard Deviation)		
	Placenta (*n* = 28)	Maternal Buccal Cells (*n* = 28)	Neonatal Buccal Cells (*n* = 28)
*BDNF*	6.92 ± 7.94	10.47 ± 10.13	7.71 ± 8.22
*CYP1A1*	19.65 ± 9.90	26.71 ± 6.92	15.23 ± 7.31
*DNMT3B*	5.02 ± 5.58	7.36 ± 11.00	7.41 ± 7.76
*Erα*	0.55 ± 1.11	5.97 ± 6.95	8.48 ± 11.34
*H19*	58.32 ± 10.57	73.60 ± 11.80	66.56 ± 16.52
*HSD11β2*	5.16 ± 4.22	0.99 ± 2.62	1.96 ± 3.60
*IGF2*	50.35 ± 8.00	39.64 ± 11.38	33.88 ± 11.03
*LEP*	65.62 ± 17.96	11.48 ± 6.06	4.93 ± 4.62
*MECP2*	7.04 ± 5.86	34.22 ± 13.88	11.44 ± 9.65
*MGMT*	0.47 ± 0.56	0.16 ± 0.30	0.33 ± 0.59
*MTHFR*	10.47 ± 3.73	17.76 ± 7.91	9.41 ± 6.88
*OXTR*	1.23 ± 1.72	1.85 ± 3.17	1.20 ± 1.28
*RELN*	2.42 ± 3.10	3.45 ± 4.14	4.02 ± 3.71
*NR3C1*	23.81 ± 18.57	5.95 ± 4.35	6.75 ± 4.74
*COMT*	30.56 ± 9.77	49.43 ± 23.67	8.32 ± 8.60
Global methylation (5-mC content)	4.90 ± 2.74	3.53 ± 2.69	3.55 ± 2.57
Global hydroxymethylation (5-hmC content)	0.38 ± 0.49	0.18 ± 0.24	0.24 ± 0.31

**Table 6:** Metals and dioxin/PCBDL placenta concentrations expressed as mean ± standard deviation (SD).

	Silver (Ag) mg/kg	Nickel (Ni) mg/kg	Vanadium (V) mg/kg	Mercury (Hg) mg/kg	Arsenic (As) mg/kg	Chrome (Cr) mg/kg	Cadmium (Cd) mg/kg	Lead (Pb) mg/kg	Dioxins (PCDD/F) + PCBDL pg TEQ/g
Placenta concentration (mean ± SD)	0.004 ± 0.007	0.03 ± 0.02	0 ± 0	0.029 ± 0.02	0.001 ± 0.003	0.09 ± 0.07	0.033 ± 0.017	0.052 ± 0.058	0.07 ± 0.11

## Data Availability

The data generated and/or analyzed during the current study are available from the corresponding author on reasonable request following approval by the Italian Ministry of Health.
